# Dietary caffeine intake is associated with favorable metabolic profile among apparently healthy overweight and obese individuals

**DOI:** 10.1186/s12902-023-01477-1

**Published:** 2023-10-20

**Authors:** Shadia Hamoud Alshahrani, Yasir A. Atia, Raheem Atiya Badir, Sami G. Almalki, Nahla A Tayyib, Sana Shahab, Rosario Mireya Romero-Parra, Mohammed Kadhem Abid, Beneen M. Hussien, Pushpamala Ramaiah

**Affiliations:** 1 Medical Surgical Nursing Department, King Khalid University, Khamis Mushate, Saudi Arabia; 2 Department of Medical Chemistry, Al-Kindy College of Medicine, University of Baghdad, University of Baghdad, Baghdad, Iraq; 3 College of Nursing, Al-Bayan University, Baghdad, Iraq; 4 Department of Medical Laboratory Sciences, College of Applied Medical Sciences, Majmaah University, Majmaah, 11952 Saudi Arabia; 5 Vice Deanship, Postgraduate Research and Scientific Studies, Faculty of Nursing, Umm Al-Qura University, Makkah, Saudi Arabia; 6 Department of Business Administration, College of Business Administration, Princess Nourah bint Abdulrahman University, P.O. Box 84428, Riyadh, 11671 Saudi Arabia; 7 Department of General Studies, Universidad Continental. Lima, Universidad Continental, Lima, Peru; 8 Department of Anesthesia, College of Health & medical Technology, Al-Ayen University, Thi-Qar, Iraq; 9 Medical Laboratory Technology Department, College of Medical Technology, The Islamic University, Najaf, Iraq; 10 Faculty of Nursing, Umm al- Qura University, Makkah, Saudi Arabia

**Keywords:** Dietary caffeine intake, Obesity, Overweight, Metabolic parameters, Glycemic status

## Abstract

**Background:**

Recent studies have revealed some conflicting results about the health effects of caffeine. These studies are inconsistent in terms of design and population and source of consumed caffeine. In the current study, we aimed to evaluate the possible health effects of dietary caffeine intake among overweight and obese individuals.

**Methods:**

In this cross-sectional study, 488 apparently healthy individuals with overweight and obesity were participated. Dietary intake was assessed by a Food Frequency Questionnaire (FFQ) and the amount of dietary caffeine was calculated. Body composition was determined by bioelectrical impedance analysis (BIA). Enzymatic methods were used to evaluate serum lipid, glucose, and insulin concentrations.

**Results:**

Those at the highest tertile of dietary caffeine intake had lower percentage of fat mass, higher fat free mass and appetite score (P < 0.05). Also, lower total cholesterol (TC) and low density lipoprotein cholesterol (LDL-c) was observed in higher tertiles of dietary caffeine intake compared with lower tertiles. In multinomial adjusted models, those at the second tertile of dietary caffeine intake were more likely to have higher serum insulin (P = 0.04) and lower homeostatic model assessment of insulin resistance (HOMA-IR) values compared with first tertile (P = 0.03) in crude model. While, in the age, body mass index (BMI), sex, physical activity, socio-economic status (SES) and energy intake –adjusted model (Model III), those at the third tertile of dietary caffeine intake were more likely to have low serum LDL concentrations [odds ratio (OR) = 0.957; CI = 0.918–0.997; P = 0.04]. With further adjustment to dietary vegetable, fiber and grain intake, those at the third tertile of dietary caffeine intake were more likely to have low systolic blood pressure (SBP), LDL and high HDL levels compared with those at the first tertile (P < 0.05).

**Conclusion:**

High intakes of dietary caffeine was associated with lower LDL, SBP, insulin resistance and higher HDL concentrations among overweight and obese individuals. However, due to observational design of the study, causal inference is impossible and further studies are warranted to confirm our findings.

## Introduction

Caffeine (3,7-dihydro-13,7-trimethyl-1H-purine-2,6-dione), is the most studied pharmacologically active substance in coffee, tea and soft drinks, cola, chocolate and cocoa [1]. From the caffeine dietary sources, coffee, is the most important source of caffeine and it is one of the world’s most popular beverages; it is estimated that four hundred billion cups of coffee are consumed each and every year [2]. Also, tea drinking is a common, popular social habit in Saudi Arabia [3]. In a systematic review of 472 articles, it was established that during the coronavirus pandemic, tea consumption clearly increased (70% versus 30%) while no clear trend in coffee consumption was observed (7 of 13 studies indicated an increase, accounting for 53.8%) [4]. Caffeine is well-known for its stimulant effects and numerous evidence has established its effects on wellbeing, happiness, energy, alertness, and sociability [5]. However, other health effects of caffeine consumption toward cardiovascular health, metabolic disorders and neurological problems have also been revealed in some of the previous studies; moderate caffeine consumption was associated with reduced risk of all-cause and cardiovascular mortality in elderly hypertensive patients [1]. Also, in a previous follow-up study, after 6 years follow-up, a 42% lower incidence of cardiovascular diseases was observed in coffee drinkers, compared to non-drinkers [hazard ratio (HR) = 0.58, 95% CI = 0.36–0.93; P _trend_ = 0.023) [6]. Also, in previous meta-analysis, coffee and caffeine intake were significantly associated with reduced incidence of type 2 diabetes mellitus (T_2_DM) [7]. In a cross-sectional study by Kim K et al. [8], habitual coffee consumption was associated with reduced risk of metabolic syndrome among Korean adults [8]. Similarly, in a meta-analysis by Shen H et al [9], total caffeine consumption was not associated with the prevalence or hepatic fibrosis of non-alcoholic fatty liver disease (NAFLD), but, regular coffee consumption significantly reduced hepatic fibrosis in patients with NAFLD. Although numerous studies regarding the health effects of caffeine on metabolic parameters are available, but, most of these studies are focused on coffee consumption and the isolate effects of dietary caffeine on metabolic parameters is evaluated in a very limited number of studies. Caffeine is consumed not only in coffee but also in soft drinks and tea and other kinds of beverages and since coffee also contains many other ingredients, caffeine and coffee cannot be considered the same [1, 10]. In obesity, most of the studies focused on the weight-reducing effects of caffeine in interventional designs and reported its positive effects on weight loss and body fat reduction [11–14]. Whereas, obesity, as a major growing health problem, is associated with numerous cardio-metabolic risk factors like dyslipidemia, metabolic syndrome, hyperglycemia and increased blood pressure [15–18]. Therefore, it is worthy to evaluate the possible beneficial effects of total dietary caffeine intake and cardio metabolic risk factors in obesity. The health effects of coffee and caffeine consumption is dose-dependent and the beneficial effects are observed in habitual and moderate consumptions but not in heavy consumption [19, 20]; it is established that caffeine intake in moderate dosages (200–300 mg) for adults is associated with reduced risk of chronic diseases like obesity and T_2_DM. Also, consumption of up to 400 mg of caffeine per day for adults and children, and 200 mg per day for pregnant and lactating women are considered safe [21, 22]. However, using more than safe doses is known to be associated with hypertension, cardiovascular disease’ risks and anxiety [21, 23]. Reduced heart rate and reduced blood pressure are reported in consumption of moderate doses of caffeine [24], while in high doses, caffeine increases the risk of hypertension [25, 26]. About physical functions, it has been shown that higher coffee consumption is strongly associated with improved physical functioning outcomes like weakness, physical frailty and muscle wasting [27–29]. Coffee consumption has been shown to reduce the prevalence of low muscle mass among 2085 adults aged 40–87 years in WASEDA’S Health Study [30]. Although, there are some discrepancies regarding this association [31]. Considering the conflicting results about the health effects of caffeine, and lack of an organized study about the possible association of the dietary caffeine intake with obesity-related metabolic parameters, in the current cross-sectional study, we aimed to evaluate the association between dietary caffeine intake and metabolic risk factors including anthropometric features, body composition, serum lipids and glycemic markers among obese individuals.

## Materials and methods

### Study population

In the present cross-sectional study, 488 randomly chosen volunteers who were overweight or obese [body mass index (BMI) > 25 kg/m^2^] and between the ages of 20 and 50 were invited by public announcements. Sampling was performed in the Nutrition and Diet centers of Riyadh Clinics. The age range was chosen to remove the possible confounding effect of menopause of women on the study variables (e.g. serum lipids, blood pressure, glycemic markers [32–34]. We excluded women who were pregnant, breast-feeding, or post menopause. Also, those who underwent bariatric surgeries, or had different types of cancer, cardiovascular diseases and diabetes mellitus were excluded.

### Demographic and dietary assessments

Demographic information were gathered through questionnaires and interviews. Data of education, employment, family size and occupation were used to estimate socio-economic status. Using the Arabic version of Depression, Anxiety, and Stress Scale (DASS)-21, the frequency of depression, anxiety, and stress-related symptoms were evaluated [35]. Visual analogue scale (VAS) was used to assess the state of the appetite [36]. Physical activity was assessed by international physical activity questionnaire (IPAQ) [37, 38].

A validated, semi-quantitative food frequency questionnaire (FFQ) with 140 food items, with acceptable validity and reliability, that was adapted for the Saudi’s general population was used to obtain data of dietary consumptions [39]. The Saudi’s household manual’s recommendations for dietary food amounts cooking yields and portion sizes were used to ask subjects about their food and beverage consumption and were converted to gram. Participants were questioned about frequency of drinking coffee or tea in the preceding year, considering a given portion size (cups per day or week or month). Caffeine intake was calculated as mg/day, from the sum of caffeine content in tea, coffee, soft drinks and chocolates.

### Anthropometric measurements

Weight and height were measured to the nearest 0.1 in kg and 0.1 cm, respectively, without shoes and with light clothes. WC was measured to the nearest 0.1 cm at the midpoint between the lowest rib margin and the iliac crest. The bioelectrical impedance analysis (BIA) method was employed by Tanita, BC-418 MA (Tanita Corporation, Tokyo, Japan) to provide detailed body composition data through the use of 8 polar electrodes in less than thirty seconds. The results of BIA includes fat mass (FM), fat free mass (FFM), and muscle mass.

### Measurement of blood biomarkers and blood pressure assessments

A trained physician used a standard mercury sphygmomanometer with an inflatable cuff (OMRON M6) to measure the subject’s blood pressure. Ten ml blood samples were obtained from all of the participants. Serum lipids and fasting blood glucose were measured with commercial kits. Serum LDL was calculated with the Friedewald Eqs. [40, 41]. Enzyme-linked immunosorbent assay (ELISA) kit was used to determine serum insulin levels (Bioassay Technology Laboratory, Shanghai Korean Biotech, Shanghai City, China). Homeostatic model assessment for insulin resistance (HOMA-IR), and the quantitative insulin sensitivity check index (QUICKI) were estimated [42].

### Statistical analysis

The Statistical Package for Social Sciences (version 23.0; SPSS Inc, Chicago IL) was used for statistical analysis. Discrete and continuous variables were reported as frequency (%), and mean ± SD. The Chi-square test and one-way analysis of variance (ANOVA) were used to assess the differences in discrete and continuousvariables across different tertiles of dietary caffeine intake, respectively. In addition, four multivariable-adjusted models of multinomial logistic regression were used to estimate odds ratios (ORs) and 95% confidence intervals (CIs) to evaluate the association between biochemical risk factors in different tertiles of dietary caffeine intake.

## Results

General demographic characteristics of study population are presented in Table 1. As shown, lower percentage of fat mass, higher fat free mass, appetite score and basal metabolic rate (BMR) were observed in higher tertiles of dietary caffeine intake (P < 0.05). No significant difference was observed for other parameters like DASS scale and socio-economic status between different tertiles of dietary caffeine intake. Table 2, represents dietary intake of calorie, macronutrients and food groups among study population. As shown, significantly higher energy, vegetable, fiber and grains consumption was observed in higher tertiles of dietary caffeine intake compared with lower tertiles (P < 0.05). No significant difference was observed for other dietary ingredients. The comparison of biochemical variables in different tertiles of dietary caffeine intake is presented in Table 3. In one way ANOVA, lower TC and LDL were observed in higher tertiles of dietary caffeine intake (P = 0.049 and P = 0.013 respectively). Although, SBP and insulin levels were reduced and HDL was increased within tertiles of dietary caffeine intake, but these changes were not statistically significant (P > 0.05). In the crude model of multinomial logistic regression (Table 4), those at the second tertile of dietary caffeine intake were more likely to have higher serum insulin (P = 0.04) and lower HOMA-IR values compared with those at the first tertile (P = 0.03). While, in the age, BMI, sex, physical activity, SES and energy intake –adjusted model (Model III), those at the third tertile of dietary caffeine intake were more likely to have low serum LDL concentrations [odds ratio (OR) = 0.957; CI = 0.918–0.997; P = 0.04]. In the fourth model, when we further adjusted the third model for dietary intake of vegetable, fiber and grain intake, being at the third tertile of dietary caffeine intake was associated with lower SBP (OR = 0.95; CI = 0.91-1.0; P = 0.05), LDL (OR = 0.95; CI = 0.90–0.99; P = 0.03) and higher HDL (OR = 1.02; CI = 1.01–1.03; P = 0.03) concentrations compared with reference category. No significant difference for other variables was observed.

**Table 1:** General demographic characteristics of study population by tertiles of DCI.

Variables		N	Mean	SD	P- value
Age (y)	1st (55.46–67.98 mg)	162	41.08	9.79	0.767
	2nd (156.43–168.04 mg)	163	40.61	8.63	
	3rd (333.94–412.12 mg)	163	40.18	9.18	
Gender [% male]	1st (55.46–67.98 mg)	162	87	66.1	0.068
	2nd (156.43–168.04 mg)	163	92	60.9	
	3rd (333.94–412.12 mg)	163	164	53.5	
BMI (kg/m^2^)	1st (55.46–67.98 mg)	162	32.47	5.52	0.690
	2nd (156.43–168.04 mg)	163	32.56	4.47	
	3rd (333.94–412.12 mg)	163	32.99	4.40	
FM (%)	1st (55.46–67.98 mg)	162	36.60	9.89	**0.010**
	2nd (156.43–168.04 mg)	163	33.98	8.99	
	3rd (333.94–412.12 mg)	163	31.63	8.18	
FFM (%)	1st (55.46–67.98 mg)	162	58.08	11.55	**0.013**
	2nd (156.43–168.04 mg)	163	63.39	12.68	
	3rd (333.94–412.12 mg)	163	64.31	12.05	
WC (cm)	1st (55.46–67.98 mg)	162	106.55	10.10	0.98
	2nd (156.43–168.04 mg)	163	107.84	9.41	
	3rd (333.94–412.12 mg)	163	107.67	8.96	
BMI (kg/m^2^)	1st (55.46–67.98 mg)	162	32.47	5.52	0.690
	2nd (156.43–168.04 mg)	163	32.56	4.47	
	3rd (333.94–412.12 mg)	163	32.99	4.40	
SES score	1st (55.46–67.98 mg)	162	9.54	2.78	0.233
	2nd (156.43–168.04 mg)	163	9.90	2.24	
	3rd (333.94–412.12 mg)	163	10.31	2.49	
DASS	1st (55.46–67.98 mg)	162	20.60	10.60	0.583
	2nd (156.43–168.04 mg)	163	18.91	11.47	
	3rd (333.94–412.12 mg)	163	20.89	12.31	
Appetite	1st (55.46–67.98 mg)	162	32.05	9.37	**0.027**
	2nd (156.43–168.04 mg)	163	32.32	8.96	
	3rd (333.94–412.12 mg)	163	35.76	8.23	
BMR (kcal)	1st (55.46–67.98 mg)	162	7365.05	1589.48	**0.032**
	2nd (156.43–168.04 mg)	163	8054.09	1485.69	
	3rd (333.94–412.12 mg)	163	8045.07	1690.04	
PA (Met. min/ week)	1st (55.46–67.98 mg)	162	1481.50	2423.68	0.092
	2nd (156.43–168.04 mg)	163	2065.30	2595.66	
	3rd (333.94–412.12 mg)	163	2737.75	4036.89	

DCI, dietary caffeine intake, BMI, body mass index, FM, fat mass; FFM, fat free mass; WC, waist circumference; SES, socioeconomic status; DASS, Depression, Anxiety, and Stress Scale; BMR, basal metabolic rate; PA, physical activity

**Table 2:** Energy, macronutrients and food groups’ intake of study population according to tertiles of DCI

Food item		N	Mean	SD	P- value
Energy (kcal)	1st (55.46–67.98 mg)	162	2708.08	948.44	< 0.001
	2nd (156.43–168.04 mg)	163	2957.24	1048.02	
	3rd (333.94–412.12 mg)	163	3393.89	1177.49	
Carbohydrate (%)	1st (55.46–67.98 mg)	162	57.21	7.03	0.537
	2nd (156.43–168.04 mg)	163	57.85	7.51	
	3rd (333.94–412.12 mg)	163	58.59	6.29	
Fat (%)	1st (55.46–67.98 mg)	162	31.94	7.20	0.946
	2nd (156.43–168.04 mg)	163	31.57	7.65	
	3rd (333.94–412.12 mg)	163	31.56	6.13	
Protein (%)	1st (55.46–67.98 mg)	162	13.41	1.82	0.870
	2nd (156.43–168.04 mg)	163	13.25	1.85	
	3rd (333.94–412.12 mg)	163	13.52	2.11	
Fruit (g/d)	1st (55.46–67.98 mg)	162	3.5	2.10	0.147
	2nd (156.43–168.04 mg)	163	4.03	2.90	
	3rd (333.94–412.12 mg)	163	4.68	3.80	
Vegetable (g/d)	1st (55.46–67.98 mg)	162	3.04	1.91	**0.003**
	2nd (156.43–168.04 mg)	163	3.89	1.99	
	3rd (333.94–412.12 mg)	163	4.42	2.54	
Fiber (g/d)	1st (55.46–67.98 mg)	162	58.78	35.14	**0.007**
	2nd (156.43–168.04 mg)	163	70.25	41.22	
	3rd (333.94–412.12 mg)	163	83.48	49.30	
Grain (g/d)	1st (55.46–67.98 mg)	162	12.37	6.67	**0.031**
	2nd (156.43–168.04 mg)	163	14.22	6.41	
	3rd (333.94–412.12 mg)	163	15.66	7.33	
Dairy (g/d)	1st (55.46–67.98 mg)	162	1.95	1.37	0.403
	2nd (156.43–168.04 mg)	163	1.99	1.20	
	3rd (333.94–412.12 mg)	163	2.23	1.34	
Meat (g/d)	1st (55.46–67.98 mg)	162	1.36	1.12	0.576
	2nd (156.43–168.04 mg)	163	1.50	1.34	
	3rd (333.94–412.12 mg)	163	1.61	1.41	
Fish (g/d)	1st (55.46–67.98 mg)	162	0.37	0.51	0.243
	2nd (156.43–168.04 mg)	163	0.24	0.30	
	3rd (333.94–412.12 mg)	163	0.32	0.42	
Poultry (g/d)	1st (55.46–67.98 mg)	162	0.69	0.67	0.592
	2nd (156.43–168.04 mg)	163	0.77	0.54	
	3rd (333.94–412.12 mg)	163	0.81	0.71	
Beans (g/d)	1st (55.46–67.98 mg)	162	0.61	0.46	0.237
	2nd (156.43–168.04 mg)	163	0.84	0.93	
	3rd (333.94–412.12 mg)	163	0.74	0.62	

P* values derived from One-Way ANOVA with *Tukey’s* post-hoc comparisons

**Table 3:** Biochemical variables in study population according to tertiles of DCI

Biochemical variables		N	Mean	SD	P- value
SBP (mmHg)	1st (55.46–67.98 mg)	162	123.75	14.48	0.728
	2nd (156.43–168.04 mg)	163	122.30	17.45	
	3rd (333.94–412.12 mg)	163	122.20	16.65	
DBP (mmHg)	1st (55.46–67.98 mg)	162	82.31	11.13	0.502
	2nd (156.43–168.04 mg)	163	82.04	12.86	
	3rd (333.94–412.12 mg)	163	80.60	11.18	
FBS (mg/dl)	1st (55.46–67.98 mg)	162	93.21	15.87	0.886
	2nd (156.43–168.04 mg)	163	92.06	14.41	
	3rd (333.94–412.12 mg)	163	93.11	25.96	
TC (mg/dl)	1st (55.46–67.98 mg)	162	192.86	37.05	**0.049**
	2nd (156.43–168.04 mg)	163	196.89	41.42	
	3rd (333.94–412.12 mg)	163	185.38	30.70	
TG (mg/dl)	1st (55.46–67.98 mg)	162	154.42	105.04	0.163
	2nd (156.43–168.04 mg)	163	160.17	96.22	
	3rd (333.94–412.12 mg)	163	137.41	75.77	
HDL (mg/dl)	1st (55.46–67.98 mg)	162	42.92	9.08	0.704
	2nd (156.43–168.04 mg)	163	43.87	9.78	
	3rd (333.94–412.12 mg)	163	43.83	9.71	
LDL (mg/dl)	1st (55.46–67.98 mg)	162	124.96	33.91	**0.013**
	2nd (156.43–168.04 mg)	163	128.93	34.24	
	3rd (333.94–412.12 mg)	163	116.62	26.48	
Insulin (mIU/l)	1st (55.46–67.98 mg)	162	17.18	12.47	0.180
	2nd (156.43–168.04 mg)	163	17.72	17.59	
	3rd (333.94–412.12 mg)	163	14.11	10.60	
HOMA-IR	1st (55.46–67.98 mg)	162	4.12	3.31	0.195
	2nd (156.43–168.04 mg)	163	4.01	3.79	
	3rd (333.94–412.12 mg)	163	3.29	2.74	
QUICKI	1st (55.46–67.98 mg)	162	0.32	0.04	0.122
	2nd (156.43–168.04 mg)	163	0.32	0.03	
	3rd (333.94–412.12 mg)	163	0.33	0.03	

DCI, dietary caffeine intake; SBP, Systolic Blood Pressure; DBP, Diastolic Blood Pressure; TC, Total Cholesterol; TG, Triglyceride; HDL-C, High Density Lipoprotein Cholesterol; LDL-C, Low Density Lipoprotein Cholesterol; HOMA-IR, Homeostatic Model Assessment for Insulin Resistance; QUICKI, Quantitative Insulin sensitivity Check Index; P-values are achieved from one-way ANOVA

**Table 4:** The odds of biochemical variables in second and third tertile of DCI versus first tertile in study population

DCI tertiles		OR	95% CI		P-value (I)	OR	95% CI		P-value (II)	OR	95% CI		P- value (III)	OR	95% CI		P- value (IV)
			LCI	UCI			LCI	UCI			LCI	UCI			LCI	UCI	
1st	1 (Ref.)																
2nd	SBP (mmHg)	0.99	0.96	1.03	0.62	0.98	0.97	1.02	0.43	0.99	0.95	1.02	0.41	0.98	0.93	1.02	0.20
	DBP (mmHg)	0.99	0.96	1.04	0.98	1.04	0.96	1.05	0.85	1.01	0.96	1.05	0.81	1.04	0.99	1.09	0.13
	FBS (mg/dl)	1.03	0.99	1.06	0.12	1.02	0.97	1.06	0.25	1.02	0.98	1.06	0.26	1.01	0.96	1.06	0.86
	TC (mg/dl)	1.02	0.98	1.02	0.83	1.01	0.98	1.03	0.89	1.03	0.98	1.03	0.81	1.01	0.99	1.02	0.81
	TG (mg/dl)	1.03	0.99	1.09	0.34	1.03	0.99	1.08	0.39	1.02	0.99	1.01	0.39	1.01	0.99	1.02	0.17
	HDL (mg/dl)	1.01	0.97	1.05	0.59	1.03	0.98	1.07	0.23	1.03	0.98	1.07	0.23	1.02	0.96	1.08	0.52
	LDL (mg/dl)	1.00	0.98	1.03	0.99	1.01	0.98	1.03	0.95	1.00	0.98	1.03	0.97	1.00	0.99	1.07	0.87
	Insulin (mIU/l)	1.18	1.01	1.39	**0.04**	1.16	0.98	1.37	0.08	1.16	0.98	1.38	0.09	1.09	0.82	1.44	0.56
	HOMA-IR	0.46	0.22	0.94	**0.03**	0.49	0.24	1.03	0.06	0.50	0.24	1.05	0.07	0.64	0.25	1.73	0.37
	QUICKI	0.99	0.98	1.20	0.53	0.98	0.55	1.71	0.38	0.98	0.98	1.01	0.44	0.98	0.98	1.22	0.47
3rd	SBP (mmHg)	1.01	0.97	1.03	0.86	0.99	0.96	1.03	0.80	0.99	0.96	1.03	0.61	0.95	0.91	1.00	**0.05**
	DBP (mmHg)	0.99	0.96	1.04	0.95	1.08	0.96	1.05	0.70	1.02	0.97	1.06	0.50	1.06	0.10	1.12	0.06
	FBS (mg/dl)	1.02	0.98	1.05	0.28	1.01	0.98	1.05	0.52	1.01	0.97	1.05	0.55	0.98	0.94	1.04	0.54
	TC (mg/dl)	1.03	0.99	1.07	0.15	1.03	0.99	1.07	0.16	1.04	0.99	1.08	0.09	0.99	0.98	1.06	0.23
	TG (mg/dl)	0.99	0.99	1.06	0.55	0.99	0.99	1.06	0.52	0.99	0.99	1.05	0.44	1.09	0.98	1.09	0.19
	HDL (mg/dl)	0.99	0.94	1.04	0.76	1.04	0.96	1.07	0.60	1.01	0.96	1.07	0.67	1.02	1.01	1.03	**0.03**
	LDL (mg/dl)	0.96	0.92	1.02	0.06	0.96	0.92	1.02	0.06	0.96	0.92	0.99	**0.04**	0.95	0.90	0.99	**0.03**
	Insulin (mIU/l)	1.09	0.93	1.27	0.30	1.06	0.91	1.25	0.45	1.06	0.90	1.26	0.46	0.94	0.72	1.24	0.67
	HOMA-IR	0.67	0.35	1.27	0.22	0.75	0.38	1.45	0.38	0.74	0.37	1.48	0.39	1.14	0.46	2.82	0.77
	QUICKI	0.99	1.09	1.02	0.37	0.98	0.93	1.03	0.48	0.94	0.51	1.73	0.59	0.98	1.10	1.02	0.97

DCI, dietary caffeine intake; SBP, Systolic Blood Pressure; DBP, Diastolic Blood Pressure; TC, Total Cholesterol; TG, Triglyceride; HDL-C, High Density Lipoprotein Cholesterol; LDL-C, Low Density Lipoprotein Cholesterol; HOMA-IR, Homeostatic Model Assessment for Insulin Resistance; QUICKI, Quantitative Insulin sensitivity Check Index; OR, odds ratio; CI, confidence interval. The multivariate multinomial logistic regression was used for estimation of ORs and confidence interval (CI). Model I: crude, Model II: adjusted for age and sex, Model III: adjusted for age, BMI, sex, physical activity, SES and energy intake. Model IV: adjusted for age, BMI, sex, physical activity, SES and dietary energy, vegetable, fiber and grain intake

## Discussion

In the current study, we observed lower body fat mass and higher fat free mass, better glycemic status and lower LDL cholesterol in higher tertiles of dietary caffeine intake. Lower fat mass and higher fat free mass in those with the highest dietary caffeine intake without any difference in BMI is attributed to the health effects of caffeine in weight regulation include increased energy expenditure and fat oxidation, inhibits phosphordiesterase [43], increase thermogenesis and fat oxidation [43, 44], reduced hormone-sensitive lipase activity [45] and increases fat oxidation via malonyl CoA and carnitine palmitoyltransferase 1 [46, 47]. Several studies have revealed the beneficial effects of caffeine on reduced fat mass accumulation; in a three phase study in adipocyte, human and animal model that was performed by Arceneaux III et al. [48], caffeine enhanced lipolysis in cultured adipocytes and acute treatment of humans with caffeine increased resting metabolic rate. There was also an increased lean mass gain concurrent with decreased fat mass gain with caffeine in the animal model that was more pronounced when it was combined with albuterol. In a randomized double blinded trial conducted by Liu AG et al. [49], the authors suggested caffeine as “a modestly effective weight loss agent that produces significant reductions in fat mass”. Although in their study, caffeine was administered as a combined 200 mg caffeine/20 mg ephedrine. It seems that dietary intake of caffeine in habitual consumption of its main sources will have weaker effects on body weight; in the study by Larsen SC et al. among 2128 participants from the Danish part of the MONICA (Monitoring Trends and Determinants in Cardiovascular Disease) cohort, no association was observed between baseline coffee consumption and 6-year changes in adiposity measurements. However, over a 6-year period, increased coffee consumption was significantly associated with reduced weight gain; although these associations were weak [31]. We observed a low TC and LDL in highest tertiles of dietary caffeine intake; the association for LDL remained significant even after adjustment for multiple confounders. Previous studies have revealed the possible role of dietary fiber, vegetable and grains in lowering serum lipids, blood pressure and glycemic markers [50, 51]; high fruit and vegetable consumption has been shown to reduce odds of high LDL concentrations to 1.00, 0.88, 0.81, and 0.75 (*P* for trend < 0.01) after adjusting for multiple confounders among apparently healthy population [51]; some of the studies have revealed that some of the water-soluble fibers, can decrease serum total cholesterol and LDL by 19% and 22% respectively [50]. Since there was a significant difference in dietary vegetable, fiber and grain intake between different categories of dietary caffeine intake in our study, we further adjusted our regression model to these variables, and while LDL reduction in the third tertile remained significant, also a reduction in SBP and an increase in HDL in the third tertile versus first tertile was observed. In fact, it seems that higher intake of dietary caffeine (e.g. 333.94–412.12 mg in our study) can have beneficial effects against serum lipids.

The health effects of caffeine on serum lipids is also investigated before; and the results are conflicting; caffeine consumption more than 200 mg per day was associated with increased serum cholesterol among women [52]. In another study, coffee consumption was in a negative association with serum TG and in a positive association with serum TC and LDL concentration [53]. In the study by Chen S et al. [1], no significant difference was observed in serum TC or TG concentrations between different categories of caffeine consumers while those with caffeine consumption greater than 200 mg/day had relatively lower HDL concentrations. A study revealed that only caffeine intake from coffee was associated with higher serum cholesterol level and this association was not observed for other dietary caffeine sources [54]. These conflicting results are due to the difference in the source of the consumed caffeine, type of it or studying the dietary caffeine or coffee consumption in different populations. Coffee consumption will exert different health effects compared with caffeine; it has been suggested that it is coffee prepared by boiling rather than other methods that has a hyper-cholesterolemic effect [55]. The suggested mechanisms are inhibition of nuclear factor-kappa B and consequent up-regulation of lipid-metabolizing enzymes, diminishing fat absorption via inhibition of gastric and pancreatic lipases, inhibition of the glucose transporters GLUT4 and SGLT1 and reduced carbohydrate oxidation [56] that could partly explain the favorable effects of dietary caffeine intake on cholesterol and glycemic markers.

**Fig. 1:**
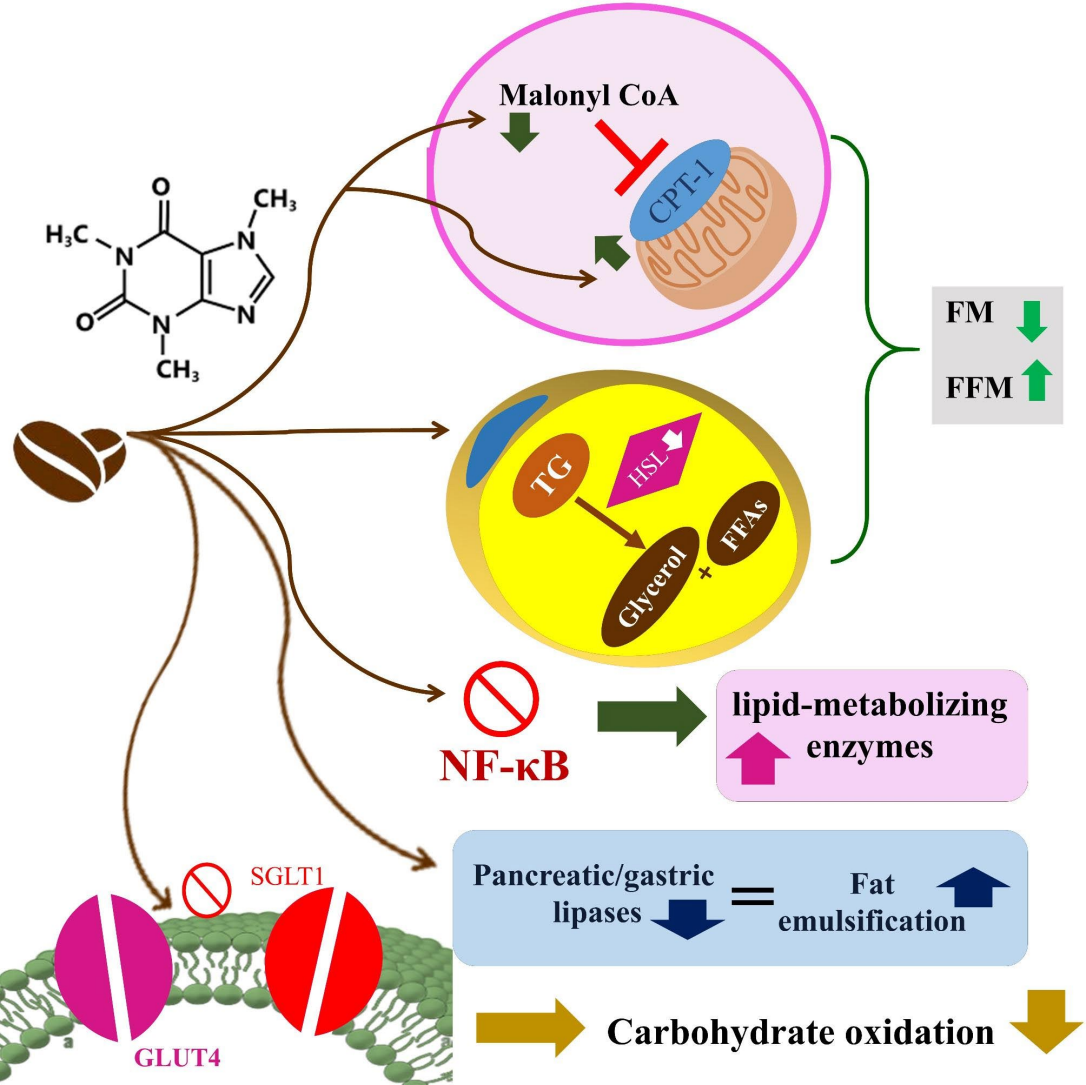
Graphical abstract of the possible mechanisms of caffeine in body fat, serum lipids and glycemic status. CPT-1, Carnitine palmitoyl transferase I; TG, triglycerides; HSL, hormone-sensitive lipase; FFAs, Free fatty acids; FM, fat mass; FFM, fat free mass, NF-κB, nuclear factor-kappa B; GLUT-4, glucose transporter 4; SGLT1, Sodium/glucose co-transporter 1

Coffee and caffeine consumption are associated with reduced incident T_2_DM in a meta-analysis of prospective studies [7]. In the Japan Public Health Center-based Prospective Diabetes study, high coffee consumption was associated with reduced fasting plasma glucose among Japanese population. The possible mechanisms of the beneficial effects of caffeine on glycemic status are increased insulin sensitivity [57] and increased adiponectin levels [58]. Intakes of caffeinated and decaffeinated coffee and caffeine were found each inversely associated with C-peptide concentration, a marker of insulin secretion in the Nurses’ Health Study [59]. In addition, caffeine might also protect against T_2_DM incidence through increasing metabolic rate and thermogenesis, stimulating fat oxidation and free fatty acid release from peripheral tissues and mobilizing glycogen in muscles [60–62]. Although, in our study, only in the crude model, the moderate intake of dietary caffeine (e.g. 156.43–168.04 mg) can reduce insulin resistance while this effect disappeared after adjustment for multiple confounders. Some of the mechanistic pathways are summarized in Fig. 1.

In the current study, we used a validated FFQ adopted for use in Saudi Arabia [39] and also the amounts of dietary caffeine is exactly measured according no caffeine intake from not only coffee but also the caffeine content in tea, soft drinks and chocolates, therefore, the results can directly be generalized into Saudi’s overweight and obese population.

The current study has some limitations; due to cross-sectional design of the current study, the causality cannot be inferred from our results. Also, we measured body composition with BIA that was not the gold standard and this may limit the interpretation of our results.

In conclusion, in the current cross-sectional study, we revealed that overweight and obese individuals at the highest tertile of dietary caffeine intake, had more favorable body composition, lower SBP, serum LDL cholesterol and lower insulin resistance. Further longitudinal and interventional studies in human models can help for generalization of our results and find the behind causality.

## Data Availability

The datasets used and/or analyzed during the current study available from the corresponding author on reasonable request.
